# Preparation and Thermal Characterization of Annealed Gold Coated Porous Silicon

**DOI:** 10.3390/ma5010157

**Published:** 2012-01-16

**Authors:** Kasra Behzad, Wan Mahmood Mat Yunus, Zainal Abidin Talib, Azmi Zakaria, Afarin Bahrami

**Affiliations:** Department of Physics, Science Faculty, Universiti Putra Malaysia, Serdang, Selangor 43400 UPM, Malaysia; E-Mails: kasra.behzad@gmail.com (K.B.); zainalat@science.upm.edu.my (Z.A.T.); azmizak@science.upm.edu.my (A.Z.); afarin.bah@gmail.com (A.B.)

**Keywords:** porous silicon, thermal diffusivity, gold, photoacoustic spectroscopy, annealing, aging effect

## Abstract

Porous silicon (PSi) layers were formed on a p-type Si wafer. Six samples were anodised electrically with a 30 mA/cm^2^ fixed current density for different etching times. The samples were coated with a 50–60 nm gold layer and annealed at different temperatures under Ar flow. The morphology of the layers, before and after annealing, formed by this method was investigated by scanning electron microscopy (SEM). Photoacoustic spectroscopy (PAS) measurements were carried out to measure the thermal diffusivity (TD) of the PSi and Au/PSi samples. For the Au/PSi samples, the thermal diffusivity was measured before and after annealing to study the effect of annealing. Also to study the aging effect, a comparison was made between freshly annealed samples and samples 30 days after annealing.

## 1. Introduction 

For several years diffusing with a gold layer has been used to control the electrical properties of silicon (Si) based devices, with several reports published on the study of gold diffusion into a silicon lattice [1,2]. Gold is highly reactive and miscible in Si, though a stable Au/Si compound does not exist, but rather a mixture of metastable [3,4] phases produced by thermal reaction and which is thought to exist under non-equilibrium conditions or in intermediate stages. The structure of metastable gold silicide is very complicated, consequently leading to various interpretations of the diffraction spectra. Investigations on gold silicide formation in thin films and the formation of metastable phases have been extensively carried out by several groups [5,6,7].

PSi is a strong light emitting material showing significant luminescence over a wide spectral range. Hence, a large amount of research work has focused on realizing porous silicon (PSi) based optoelectronic devices, such as light emitting diodes (LED) [8], waveguides [9], optical filters [8], thermal isolators [10], photovoltaic diodes [11], and different types of sensors [12]. The Au/PSi structure can be used as a hydrogen fuel cell or for other applications that require tuneable and lower thermal diffusivity than silicon. Photoluminescence [13], electrical [14,15], optical [16] and other properties of gold coated porous silicon (Au/PSi) have been reported to date; however, no reports on the thermal properties currently exist. 

Here, we report a study on the structural and thermal properties of Au/PSi with different porosities annealed at different temperatures, including an investigation on the aging effect of all samples after 30 days. The samples were grouped in four sets and each set was made porous with six different etching times. The current density was fixed for all samples at 30 mA/cm^2^. Then an Au layer (50–60 nm) was coated on all samples at room temperature. The eutectic temperature of the Au-Si system is known as 365 °C from the phase diagram [17], so the above mentioned sets were annealed from 300 to 800 °C for one hour under Ar flow to cover the temperature range below and above the eutectic temperature. So the difference between sets can be considered as the annealing temperatures. As photoacoustic spectroscopy (PAS) is an accurate and non-destructive method, we chose it to measure the TD. 

## 2. Results and Discussion

Sample thickness and porosity were calculated by the gravimetric method [18]. The samples were weighed before anodization (m_1_), immediately after anodization (m_2_), and after dissolution of the porous silicon layer in 1 M, NaOH aqueous solution (m_3_). The porosity and thickness respectively are given by the following equations:

(1) $$P\left(\%\right)=\frac{m_{1}-m_{2}}{m_{1}-m_{3}}\times 100$$

(2) $$d=\frac{m_{1}-m_{3}}{\rho \;S}$$

where ρ is the Si density and *S* is the anodised surface. The thickness of the PSi layers was also rechecked by Ambios Technology, a stylus profilometer, XP-200. 

**Figure 1:**
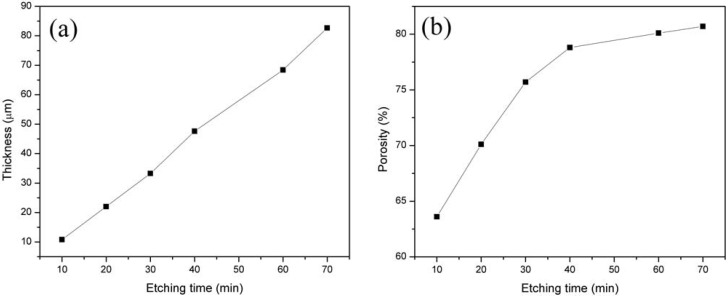
(**a**) Thickness as a function of etching time; (**b**) Porosity as a function of etching time.

By using Equations 1 and 2 after measuring the mass of the samples, the density of substrate (*ρ*) and the anodized area (S), the porosity percentage and the thickness of the layer were calculated. The variation in porosity and thickness are shown in Figure 1. The thickness of the layers rises with increasing etch time. The average thickness of the PSi layers grows roughly linearly with etching time. The thickness of these samples was also measured with the stylus profilometer. The results are in a good agreement with the values in Figure 1(a). Notably, the porosity initially increases rapidly and levels off at approximately 79%. 

**Figure 2:**
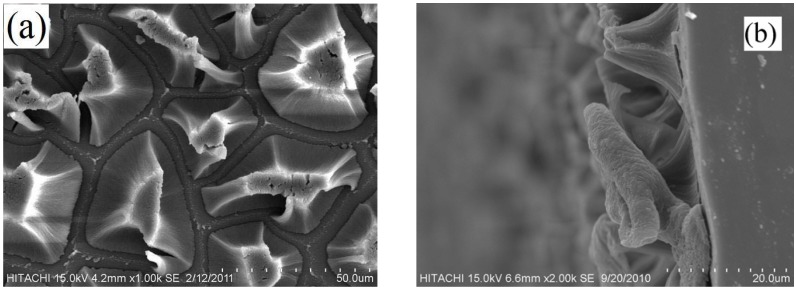
(**a**) SEM image of a PSi layer; (**b**) Cross sectional view of a PSi layer.

For structural characterization, the PSi layers were examined with scanning electron microscopy (SEM) before and after gold deposition. These images were carried out using a Hitachi, SN3400. SEM images show the PSi layer prepared on a Si substrate. The silicon structures were seen clearly on the top view in Figure 2(a), and cross sectional view in Figure 2(b). The top view shows that the size of the silicon structures are between 30–40 µm and the cross sectional view shows that the thickness of the layer for this sample is roughly around 20 µm.

**Figure 3:**
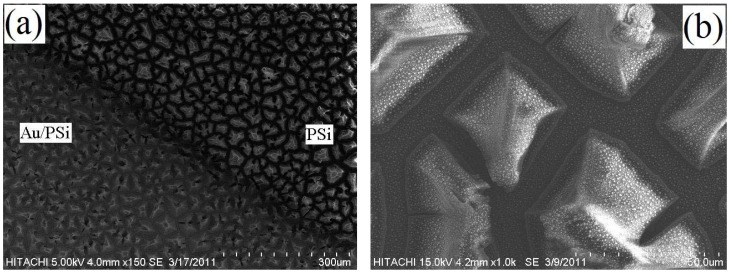
(**a**) SEM image of a PSi sample with Au deposition on a part of the surface; (**b**) image of Au deposited PSi layer after annealing.

Figure 3(a) shows a PSi sample with half of the surface uniformly deposited with an Au thin film. According to Figure 3(b), a uniform dispersion of island-like Au particles, see white dots as shown by arrow, agglomerate with a SiO_x_ matrix. This forms because of the low kinetic energy gained by sputtered Au atoms at room temperature, which lead to a slow diffusion rate and mobility [19,20].

To describe the results of the PAS measurements we refer to the thermal piston in terms of the RG theory, developed by Rosencwaig and Gersho [21]. They believed the source of the acoustic signal in the cell is caused by the periodic heat flow of the solid to the ambient gas as the solid is heated by the modulated monochromatic beam. The pressure fluctuations are described as:

(3) δ p=γ p0I0(αgαs)12exp j(ωt−π2)/2πT0lgκsf sinh(lsσs)

where γ is the air specific heat ratio, *P_o_* the ambient pressure, *I_0_* the incident light beam intensity, *f* the chopping frequency; and *l_i_*, *k_i_*, and α_i_, are the thickness, thermal conductivity and TD of material *i,* respectively. The subscript *i* can be both s and g, representing the sample and gas, respectively. TD (α_i_) is the rate of propagation of a temperature pulse in a material between two points. Thermal diffusivity defines as $\alpha =k/\rho \;C_{v}$, where α_i_ is TD, *k* is thermal conductivity, *ρ* is density, and *C_v_* is the specific heat of the sample. The complex thermal diffusion coefficient of material *i* is defined as [21]:

(4) σi=(1+j)ai, ai=1/μi=(π f/αi)12

where μ_i_ is the thermal diffusion length. Particularly, for a thermally thick sample ($l_{s}\sigma_{s}>>1$), PA amplitude (S) varies as:

(5) S=Afexp(−bf) , b=(π ls2/αs)12

For measuring the TD of samples (α_s_) the PA signal is plotted versus the chopping frequency. By fitting the PA signal to Equation 6 in the thermally thick area, the TD value can be derived from *b* as the *l_s_* is measured with a micrometer screw gauge. The TD value was also obtained from the characteristic frequency (*f_c_*), which is the modulation frequency when it passes from a thermally thin to thermally thick regime and the thermal diffusion length is equal to sample thickness, *i.e.*, $f=f_{C}\;,\;\mu_{S}=l_{S}$ [22]. In this method the characteristic frequency (*f_c_*) has been found from ln(PA signal) versus chopping frequency graph. The TD can be calculated using the Equation 6.

(6) lS=(αS/π fC)12 , αS=π fC lS2

**Figure 4:**
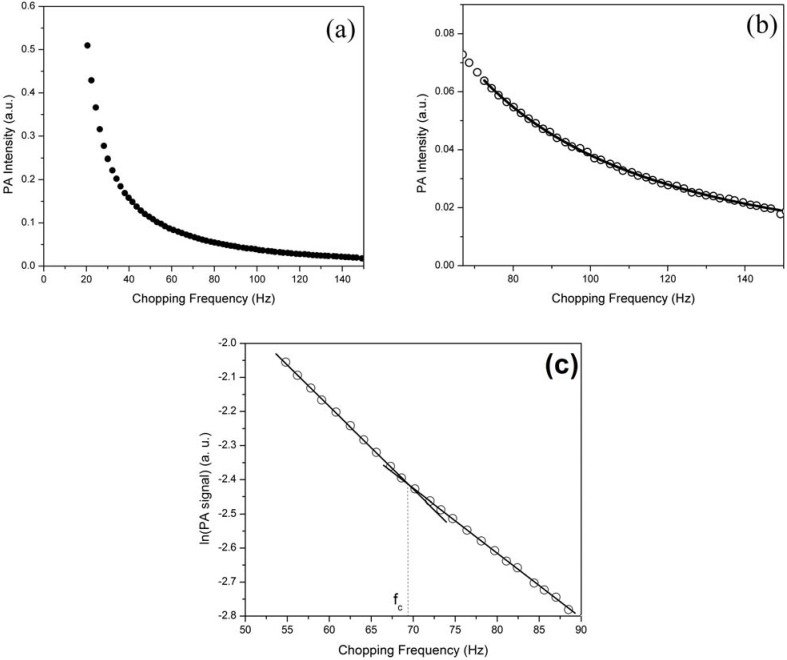
(**a**) PA spectra for a sample under a chopping frequency; (**b**) PA signal versus chopping frequency in the thermally thick regime. The solid curve represents the best fit of the data to Equation 5 in this area; (**c**) ln(PA signal) versus chopping frequency to find the characteristic frequency (*f_c_*).

Figure 4(a) shows the PA signal of the PSi/Si sample at a modulation frequency between 15 to 150 Hz. At low frequency, the thermal diffusion length is larger than the thickness of the samples’ so-called thermally thin regime (*µ_s_ > l_s_*). By increasing the frequency to a certain point, the thermal diffusion length will equal the sample thickness; this value of the frequency corresponds to the characteristic frequency (*f_c_*). By increasing the frequency, it changes to the thermally thick regime [23]. Therefore, the PA signals were fitted with Equation 5 only in the thermally thick area (*µ_s_ < l_s_*) as shown in Figure 4(b). The ln(PA signal) versus chopping frequency, Figure 4(c), shows a distinct change in slope at the frequency (*f_c_*) at which crossover takes place. The TD value was calculated for all samples by finding the characteristic frequency using Equation 6, and the values were also confirmed by this method.

Before characterising the PSi samples, the optical setup and measuring procedures were tested with a c-Si wafer and the TD values for the four samples were found between 0.84–0.90 cm^2^/s, in good agreement with values in the literature [24,25,26,27]. This measurement was done for silicon and Au/PSi before and after annealing, and also PSi before annealing, in order to measure and trace the variation of TD in the different conditions of this study.

**Figure 5:**
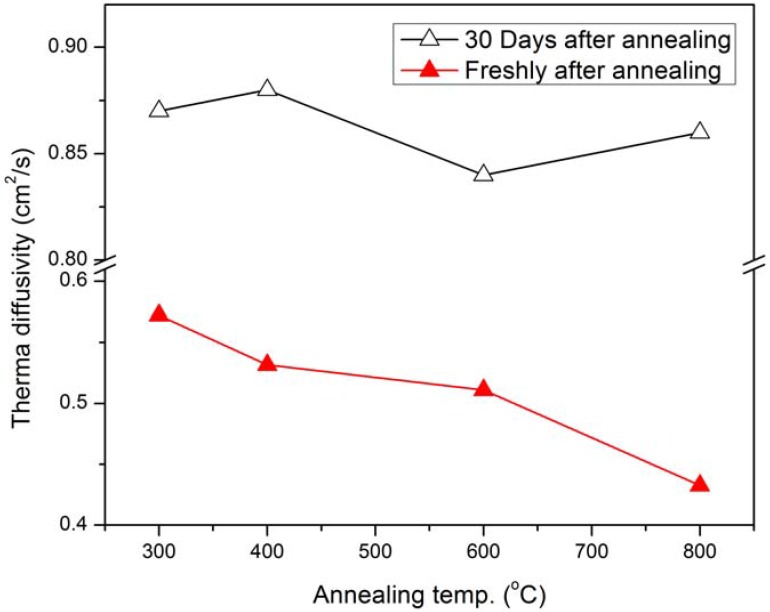
The TD of the Si for different annealing temperatures for freshly annealed and 30 days after annealing.

Figure 5 shows the TD values for silicon annealed at different temperatures. These results were taken freshly after annealing and 30 days after annealing. It is clear that in the first set, the TD of silicon decreases with increasing temperature, but returns to its primary value over time, and shows these values reach 0.84–0.88 cm^2^/s 30 days after annealing. 

**Figure 6:**
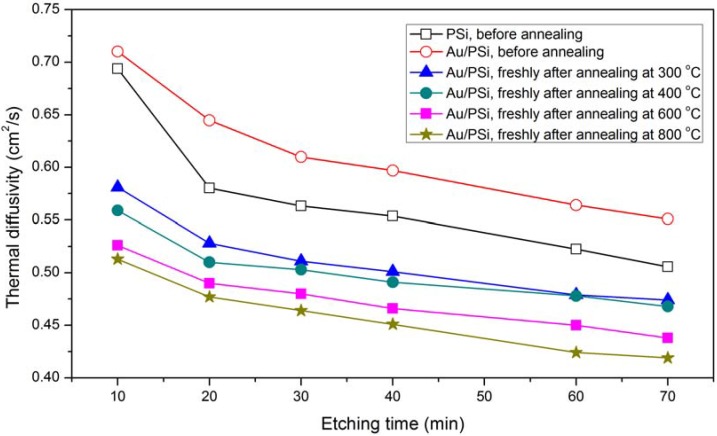
Variation of the TD with etching time for all samples. For annealed samples, it was measured exactly after cooling.

The variations in the TD value of the PSi and Au/PSi samples before and after annealing are compared in Figure 6 and the values are also shown in Table 1. It is clear that the TD increases gradually by depositing an Au layer on PSi, and then decreases with increasing annealing temperature. Also it decreases with increasing porosity. This value was measured also for the same samples after 30 days and will be described later.

**Table 1:** Comparison of the measured values for the TD (cm^2^/s).

Sample	Etching time (min)					
	10	20	30	40	60	70
**PSi**	0.693	0.580	0.563	0.553	0.522	0.505
**Au/PSi**	0.710	0.644	0.609	0.596	0.564	0.551
**Au/PSi (at T = 300 °C)**	0.581	0.528	0.511	0.501	0.479	0.474
**Au/PSi (at T = 400 °C)**	0.559	0.510	0.503	0.491	0.478	0.468
**Au/PSi (at T = 600 °C)**	0.526	0.490	0.480	0.466	0.450	0.438
**Au/PSi (at T = 800 °C)**	0.513	0.477	0.464	0.451	0.424	0.419

From results of PSi samples, a regular physical trend emerges from the behaviour of signal amplitude: with increasing modulation frequency, and therefore decreasing thermal diffusion length (µ_s_) in the sample, the surface layer has a stronger effect on the thermal transport, and the sample surface temperature progressively increases, as a consequence of the lower thermal conductivity of the layer with respect to that of the Si substrate. The decrease in the TD with increased porosity is a consequence of the decrease in the mean free path, due to the phonon confinement in the crystallites. When the phonon mean free path is less than the crystallite size, phonons are confined, and so the TD is reduced [28,29]. Increasing the TD by depositing an Au layer is due to the additional metal layer itself; with a higher TD than for PSi without an Au layer on the semiconductor substrate. In the next step, annealing the gold coated porous silicon (Au/PSi) samples caused these values to drop slightly, with increased temperature, in the range of the porosities examined. The decrease in the TD means that the thermal conductivity was decreased in the samples that are related to the thermal carrier or media. The thermal carriers are not affected by temperature in this range (300–800 °C), so the decrease in the TD is due to the temporarily deformation of the Si lattice.

The TD was measured for Au/PSi samples 30 days after annealing and was compared with the freshly annealed samples. 

**Figure 7:**
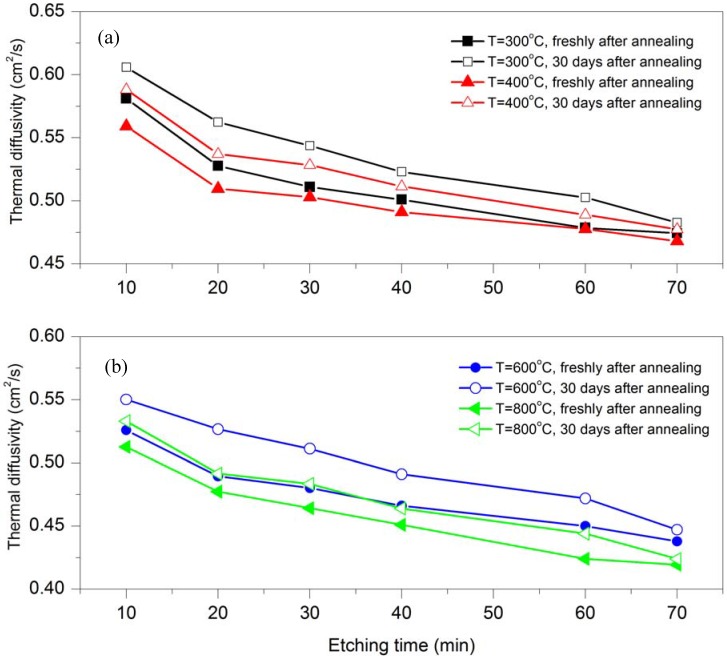
Variation of the TD with etching time for Au/PSi samples fresh after annealing and 30 days after annealing at different temperatures (**a**) T = 300 and 400 °C; (**b**) T = 600 and 800 °C.

Figure 7 shows four graphs related to different annealing temperatures at 300 °C, 400 °C, 600 °C and 800 °C. Each graph compares the TD for freshly annealed samples and the same samples after 30 days. All four graphs show that the TD decrease on raising the etching time for freshly annealed samples and for samples 30 days after annealing, but the TD for the samples 30 days after annealing increases over time. It is defined as an aging effect in that the TD gradually increases around 2–6% after 30 days. The aging effect is due to a diminished effect of the annealing on lattice deformation.

## 3. Experimental Section 

### 3.1. Sample Preparation

All samples were formed on p-type silicon single crystal wafers of 520 μm thickness and resistivity 1–10 ohm·cm, polished on one side. The wafers were cut into rectangular pieces, with the typical area of the pieces being approximately 5 cm^2^. To provide a uniform current distribution across the surface, an aluminium layer was deposited on the back side of all samples. Each sample was placed on the bottom of a cylindrical Teflon cell and fixed with an aluminium plate as a backing material. The cell consisted of two electrodes, a Si wafer as anode and a platinum rod as cathode, the cathode placed perpendicular to the anode surface. All samples were obtained by varying the etching time, and a constant concentration of HF (48–50%), ethanol (99.90%) and distilled water solution in a volumetric ratio (HF:C_2_H_5_OH:H_2_O) of 1:2:1 was used. Electric current was supplied with an ADCMT 6,243 DC voltage/current source/monitor. Our samples in this study were prepared with 30 mA/cm^2^ fixed current density and 10, 20, 30, 40, 60, and 70 min anodization times. After anodization a thin semi-transparent Au layer (50–60 nm) was deposited on all samples at room temperature, using a VG Microtech SC7640 sputter coater. The prepared samples were then annealed for one hour at 300, 400, 600 and 800 °C using a tube furnace under argon flow at atmospheric pressure. There are four sets of Au/PSi samples annealed at four different annealing temperatures. Each set consists of one piece of Si wafer that was annealed with the others to use as a reference sample.

### 3.2. Characterisation 

**Figure 8:**
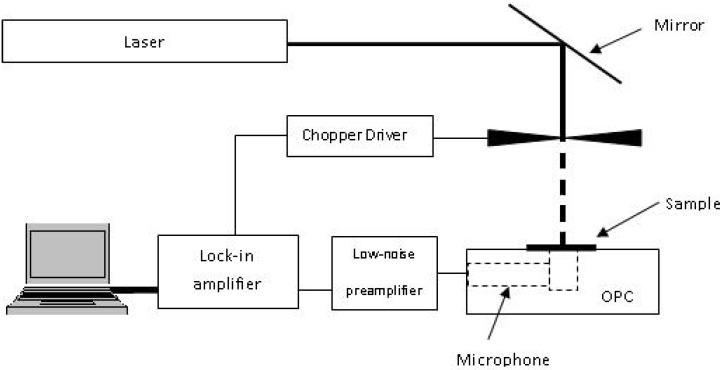
Schematic diagram of PAS setup.

The photoacoustic spectroscopy (PAS) setup consisted of a light source, detector, and data analysing system. A Melles Griot HeNe laser of 632.8 nm at a power of 75 mW was used as a light source, and was modulated by a Stanford Research Systems optical chopper SR540. A handmade open photoacoustic cell (OPC) was used as a detector. A Stanford Research Systems low-noise preamplifier, SR560, was used to amplify the very weak output signal from OPC, which was sent to a Stanford Research Systems lock-in amplifier SR530. A program written in Lab VIEW was used to control the system and collect the data from the lock-in amplifier, via a GPIB bus, Figure 8. The photoacoustic (PA) signal was obtained with chopping frequencies in the range of 10–150 Hz. 

## 4. Conclusions 

To conclude, the PAS technique has been used to study the thermal properties of the samples before and after annealing. The Si samples have different TDs based on annealing temperatures and after 30 days the TD of all silicon samples return to their initial values. For PSi/Si samples the layer thickness and porosity increased with etching time and the TD was significantly smaller than that of the Si samples. It decreased with increasing porosity caused by a decrease in the mean free path due to the phonon confinement in the crystallite. The Au/PSi samples show gradually higher values for TD, in the range of porosities examined, because of the deposited metal layer. By annealing the Au/PSi, white spots of gold islands were observed on the PSi surface. The Au was pulled together from the nearest surface to form gold clusters after annealing. In this case, the TD for freshly annealed samples was even smaller than that of the PSi/Si samples and decreased on raising the annealing temperature. Additionally, for the annealed Au/PSi samples, the TD values rose slightly after thirty days. Overall, the TD was decreased by raising the annealing temperature and thus inducing deformations in the lattice. Also aging effect showed a small increase in the TD over time due to the diminished effect of the annealing in the lattice deformation. This indicates that the TD of all samples can be controlled by changing their porosities and annealing temperatures, and then operated as tuneable thermal insulators in silicon based devices or hydrogen fuel cells with the aging effect consideration.
